# Natural Selection for Operons Depends on Genome Size

**DOI:** 10.1093/gbe/evt174

**Published:** 2013-11-06

**Authors:** Pablo A. Nuñez, Héctor Romero, Marisa D. Farber, Eduardo P.C. Rocha

**Affiliations:** ^1^Instituto de Biotecnología, Instituto Nacional de Tecnología Agropecuaria (CICVyA-INTA), Buenos Aires, Argentina; ^2^Laboratorio de Organización y Evolución del Genoma, Dpto. de Ecología y Evolución, Facultad de Ciencias/CURE, Universidad de la República, Uruguay; ^3^Institut Pasteur, Microbial Evolutionary Genomics, Paris, France; ^4^CNRS UMR3525, Paris, France

**Keywords:** operons, prokaryotes, evolution

## Abstract

In prokaryotes, genome size is associated with metabolic versatility, regulatory complexity, effective population size, and horizontal transfer rates. We therefore analyzed the covariation of genome size and operon conservation to assess the evolutionary models of operon formation and maintenance. In agreement with previous results, intraoperonic pairs of essential and of highly expressed genes are more conserved. Interestingly, intraoperonic pairs of genes are also more conserved when they encode proteins at similar cell concentrations, suggesting a role of cotranscription in diminishing the cost of waste and shortfall in gene expression. Larger genomes have fewer and smaller operons that are also less conserved. Importantly, lower conservation in larger genomes was observed for all classes of operons in terms of gene expression, essentiality, and balanced protein concentration. We reached very similar conclusions in independent analyses of three major bacterial clades (α- and β-Proteobacteria and Firmicutes). Operon conservation is inversely correlated to the abundance of transcription factors in the genome when controlled for genome size. This suggests a negative association between the complexity of genetic networks and operon conservation. These results show that genome size and/or its proxies are key determinants of the intensity of natural selection for operon organization. Our data fit better the evolutionary models based on the advantage of coregulation than those based on genetic linkage or stochastic gene expression. We suggest that larger genomes with highly complex genetic networks and many transcription factors endure weaker selection for operons than smaller genomes with fewer alternative tools for genetic regulation.

## Introduction

Most genes in the genomes of prokaryotes are expressed in polycistronic units called operons. The operon model was published more than 50 years ago and had a key role in the development of molecular biology as it highlighted the importance of gene expression regulation (Jacob and Monod 1961). Operons start with a transcription promoter, include between two and more than a dozen genes (average ∼3 genes), and end in a transcription terminator (Zheng et al. 2002; Lawrence 2003). The presence of alternative promoters and attenuators can lead to heterogeneous transcription rates within operons. Furthermore, mRNA and proteins in prokaryotes have very different half-lifes (7 min and ∼24 h, respectively, in both *Escherichia coli* and *Mycoplasma pneumoniae*; Koch and Levy 1955; Selinger et al. 2003; Maier et al. 2009; Yus et al. 2009). This leads to poor correlations between transcription rates and protein concentrations (Maier et al. 2009). Nevertheless, genes in the same operon are expressed at more similar rates than random pairs of genes (Sabatti et al. 2002; Price et al. 2006).

Operons are highly enriched in genes encoding related functions. Accordingly, operons often include genes encoding enzymes of consecutive steps in metabolic pathways (Zaslaver et al. 2006) or genes encoding interacting proteins (Mushegian and Koonin 1996; Huynen et al. 2000). Conservation of a gene in an operon is thus a strong indication of functional neighborhood and can be used to make functional inferences (Overbeek et al. 1999; Moreno-Hagelsieb and Janga 2008). Proteins often participate in several processes. Hence, the presence of a gene in an operon, and the closeness between operons, reflects a compromise between the different processes (Yin et al. 2010). Operon structure is thus a key variable to understand the regulation of gene expression in prokaryotes. Operons are much more conserved than expected given rearrangement rates (de Daruvar et al. 2002; Rocha 2006a; Moreno-Hagelsieb and Janga 2008), presumably because of the advantages of such genetic organization (Lathe et al. 2000; Omelchenko et al. 2003; Price et al. 2006).

A number of models aim at explaining the advantages of the organization of genes in operons. Some models propose that tight genetic linkage favors physical clustering of functional neighbor coevolving genes (genetic linkage models). Indeed, coadaptive changes in interacting genes are less likely to be broken by recombination if genes are closely spaced (recombination model; Stahl and Murray 1966). It is unclear if this hypothesis is compatible with the typically low recombination rates found in bacteria (Vos et al. 2009), and with the short size of recombination tracts (typically smaller than a gene; Kennemann et al. 2011). Tight linkage between genes under strong selection could also diminish the deleterious effects of large genetic deletions, which are very frequent in Prokaryotes, by reducing their pleiotropic effects and protecting functions under very strong selection (persistence model; Fang et al. 2008). These models are plausible, but the formation of operons requires some additional explanation because genes do not have to be cotranscribed to be under tight linkage. As an example, the yeast *Saccharomyces cerevisiae* shows coevolution of gene order and recombination rates leading to linkage between essential genes even though it practically lacks operons (Pal and Hurst 2003).

Horizontal gene transfer drives the expansion of the gene repertoires of prokaryotes (Ochman et al. 2000; Gogarten et al. 2002; Treangen and Rocha 2011). Genes encoding functions under constant selection—persistent genes—are stably maintained in genomes for long periods of time. This trend is particularly strong for genes encoding essential functions. Inversely, accessory genes are frequently gained and lost (Medini et al. 2005). According to the selfish model, operons form and persist because they facilitate the horizontal transfer of coregulated functional modules (Lawrence and Roth 1996). Such a set of functionally neighbor, coregulated genes is more likely to be functional in the new genetic background and thus be kept by natural selection in the new genome. However, essential genes are more clustered than the average gene and are more often in operons (Pal and Hurst 2004). Accordingly, new operons do not overrepresent horizontally transferred genes and often include essential or persistent genes (Price, Alm, et al. 2005). Interestingly, the most frequently clustered genes in genomes are either present in nearly all or in very few genomes in a clade (Fang et al. 2008). Thus, the domains of application of the selfish model and the other linkage-based models might be different. The latter could apply to the genes that persist for long periods of time in prokaryotic lineages whereas the selfish model could explain why accessory genes with high rates of transfer and loss are often found in operons.

Recent works suggest that noise minimization drives operon formation and within operon organization (Swain et al. 2002; Swain 2004; Sneppen et al. 2010; Ray and Igoshin 2012; stochastic expression models). In these models, cotranscription lowers the cost of stochastic gene expression because it synchronizes the different components of the same functional module thus minimizing shortfall or waste. Translational coupling, the interdependence of translational efficiency of genes encoded within the same operon, might also favor similar expression levels for proteins expressed from the same operon (Lovdok et al. 2009). Noise in gene expression decreases rapidly with increasing level of expression (Elowitz et al. 2002) and should be particularly relevant for lowly expressed (LE) genes in small cells such as the ones of most bacteria. Cotranscription places a series of genes under the control of a single regulatory region. This single region could be more efficiently selected for adaptive changes—relative to the set of promoters required to regulate transcription of monocystronic units—favoring more efficient regulation of expression levels (Price, Huang, et al. 2005; promoter sharing model). Genomes structured in operons have fewer target sites for deleterious mutations in regulatory regions. Hence, operons could result from selection to minimize the deleterious impact of these mutations. Accordingly, it has been proposed that reduction of effective population sizes, as observed in many eukaryotes, leads to operon disruption (drift model; Lynch 2006). This highlights the expected positive association between effective population size, through its effect in the efficiency of natural selection, and operon conservation.

The study of the formation and maintenance of operons is important to establish the evolutionary mechanisms shaping the structure of prokaryotic genomes and their regulatory networks. Unfortunately, the direct test of these models requires the quantification of parameters such as effective population sizes, rates of horizontal transfer, and selective coefficients for operons formation and change. These are notoriously difficult to estimate in prokaryotes. One variable that might be useful in this framework is genome size, which in bacteria varies from 0.14 Mb to more than 13 Mb (Schneiker et al. 2007; McCutcheon and von Dohlen 2011). Bacterial genomes are tightly packed with genes. Hence, variations in genome size correspond to proportional variations in the number of genes in the genome (Mira et al. 2001). These variations affect the classes of functional repertoires of the cell at very diverse levels (Boussau et al. 2004). Smaller genomes tend to correspond to obligatory mutualists or pathogens and arise by reduction of larger genomes of free-living bacteria (Moran 2003; Klasson and Andersson 2004; Moya et al. 2009). Large bacterial genomes correspond to species that are able to tackle a large number of environmental stimuli and sometimes encode complex developmental processes such as multicellularity (Schneiker et al. 2007). Of particular relevance for the evolution of operons is the observation that the number of transcription factors increases more than linearly with genome size (van Nimwegen 2003; Konstantinidis and Tiedje 2004). Large bacterial genomes encode many transcription factors, whereas many of the smallest genomes encode none (Minezaki et al. 2005). Genome size is also correlated with the strength of purifying selection suggesting that it is a proxy for effective population size (Kuo et al. 2009). Finally, genome size is the net result of accretion events, notably horizontal gene transfer, and genetic deletions (Lawrence et al. 2001). Hence, larger genomes are expected to have acquired genes by horizontal transfer at much higher rates than small genomes.

As genome size is correlated with the number of genes encoding transcription factors, with effective population size and with the frequency of horizontal gene transfer, it is a key amenable variable to study the evolution of operons. Yet, previous results showed equivocal trends. A seminal analysis of 26 bacterial genomes showed a negative correlation between operon and genome sizes (Cherry 2003). Accordingly, the small genomes of *Buchnera aphidicola* and *M. pneumoniae* have long operons (Guell et al. 2009; Yus et al. 2009; Brinza et al. 2010). However, other small genomes, for example, *Borrelia spp*., have short operons (Zheng et al. 2002). Operon detection methods use, amongst other information, the length of intergenic spacers to distinguish between intra- and interoperonic spacers (Salgado et al. 2000; Chuang et al. 2012). Hence, the use of the number of operons as a proxy of selection on operon conservation could be affected by the covariation of genome size and the length of intergenic spacers if operon detection is made with state-of-the-art methods. Pairs of intraoperonic genes are much more conserved than pairs of interoperonic genes (Rocha 2006a). A methodological artifact leading to spuriously large operons in compact genomes will lead to lower operon conservation in these genomes because the false putative “operons” are less likely to be conserved. As a result, while errors in operon annotation in compact genomes may lead to an overestimation of the selection acting upon large operons in these genomes, they will also lead to an underestimation of operon conservation. Operon conservation between species is also a more accurate and natural measure of the selection imposed on operons than other simple traits such as operon length and operon number. We therefore decided to analyze operon conservation as a function of genome size.

To examine the organization and distribution of operons, we analyzed three different clades: α-proteobacteria, β-proteobacteria, and Firmicutes. Analyzing different clades enabled us to inspect independent processes of genomic evolution. These clades, in particular, include many sequenced genomes and a broad range of genome sizes (from: 0.14 to 9.10 Mb in α-proteobacteria; 0.2 to 9.73 Mb in β-proteobacteria; and 1.42 to 7.18 Mb in Firmicutes; supplementary fig. S1, Supplementary Material online). In this study, we first analyzed general features of operon organization in each of the three clades. Next, we compared the operon structure of each genome with that of *E. coli*. As we analyzed each clade separately, *E. coli* is always an outgroup relative to the other genomes. Finally, we studied the variation in operon conservation as a function of genome size and relevant biological traits such as essentiality, gene expression and protein concentration.

## Materials and Methods

### Data

Complete genome sequences and annotations were downloaded from NCBI GenBank (ftp://ftp.ncbi.nih.gov, last accessed November 20, 2013; supplementary table S1, Supplementary Material online). We excluded pseudogenes and genes with internal in-frame stop codons. All the predicted operons for these organisms were downloaded from the Prokaryotic Operon DataBase (ProOpDB; http://operons.ibt.unam.mx/OperonPredictor/, last accessed November 20, 2013), which claims to be the most accurate method available (Taboada et al. 2012). As different operon predictions may differ in a number of cases (Brouwer et al. 2008), we also calculated all results using the operons predicted in the Database of prOkaryotic OpeRons (DOOR) at http://csbl1.bmb.uga.edu/OperonDB (last accessed November 20, 2013; Mao et al. 2009; supplementary text S1, Supplementary Material online). We analyzed *Escherichia coli* K-12 MG1655, 102 α-Proteobacteria, 63 β-Proteobacteria, and 124 Firmicutes. To avoid overrepresentation of highly similar organisms, those above 97% in 16SrRNA identity were clustered and the largest genome was selected for this study. We did not include Tenericutes, the basal branch of Firmicutes, in our study because their genomes are always small and evolve fast. Their inclusion would lower the accuracy of the method to detect homology and might affect our results. For the three clades, we included in our study all species with complete public annotated genome sequences for which there were whole-genome operon predictions in both databases.

### Identification of Orthologs

We identified for each genome the genes that were orthologous to the ones of *E. coli* K-12 MG1655 using reciprocal best hits. For every gene, we conducted a FASTA search in the other genome and vice versa. We retained the ten best hits for every gene. For these ten hits, we constructed exact pairwise alignments using the BLOSUM62 matrix and the Needleman–Wunsch algorithm where we did not penalize gaps at the edge of smaller sequences, that is, end-gap free alignment following (Erickson and Sellers 1983). We considered as putative orthologs the reciprocal best hits with more than 38% similarity and less than 30% difference in protein length.

### Operon Gene Pairs

The operon structure of a given genome was analyzed as a succession of intraoperon gene pairs (OGP). Starting from the operon prediction data of a given genome, the OGP were constructed for each pair of adjacent genes within the operon (there are [*k* − 1] adjacent gene pairs for a *k*-gene operon). For example, a set of adjacent genes ABC will be decomposed in two pairs: AB and BC. Under a null model of neutral evolution of operons, the individual OGPs are statistically independent, because disruption of the adjacency between B and C will not affect the adjacency between A and B. Deletion of B leads to exclusion of the two pairs from further analysis. We made a test to check that counting a gene twice—its two pairs—is not affecting our results under a selection model where disruption of an operon between AB might lead also to decrease selection for BC. We made a complementary set of the operon conservation analyses (below) with a data set where each gene is only counted once (one of the pairs is randomly discarded). Despite the fewer data points (decreasing the power of the test), we find very high correlations between the analysis with the original and the restricted data sets (all correlations larger than 0.9, see supplementary table S2 [Supplementary Material online] for a comparison). The analysis of independent OGP also avoids overrepresenting large operons. For example, we analyze two OGP in 3-gene operons and 9 OGP in 10-gene operons. If all pairwise analyses within the operon were done, there would be three comparisons for 3-gene operons and 45 comparisons for 10-gene operons. Our method assures that only one OGP is affected by one given rearrangement independently of operon size.

### Operon Conservation

We used the conservation of OGP as a measure of operon conservation between a given focal genome “A” and *E. coli* K-12 MG1655. First, from all intra-OGPs in *E. coli* K-12 MG1655, we selected those having orthologs in genome A (OGP_A_). We then identified within the intra-OGPs in the genome A those for which the orthologs in *E. coli* K-12 MG1655 were also in the same operon (OGP_A,EC_). Finally, we calculated the operon conservation index for A (OCI_A_) as follows:

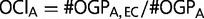

We also computed OCI values for subsets of pairs of genes according to the variables of interest such as essentiality, gene expression, and protein concentration (supplementary table S1, Supplementary Material online).

### Analysis of Expression Levels

To estimate *E. coli* K-12 MG1655 mRNA levels per cell, we used four public transcriptomic data sets (Allen et al. 2003; Corbin et al. 2003; Covert et al. 2004; Lu et al. 2007). Data obtained from different experiments were normalized. For this, we selected the genes that were assayed in all experiments (ubiquitous genes). We computed the average values for these genes in each experiment. If in experiment A the average is X_A_ and in experiment B it is 1.3*X_A_, then 1.3 is the multiplicative factor that has to be applied to the values in A so that these genes can be compared with the ones of B. The ratio of the averages between experiments gives then the multiplicative factors that allow us to normalize the experiments so that we can compare them directly. In the earlier example, a gene z assayed in A and B has a normalized value of (z_A_ + z_B_/1.3)/2. To estimate the mRNA levels of each OGP, we averaged the normalized values of both genes. Then, we used the median of this distribution (supplementary fig. S2, Supplementary Material online) to divide the set of genes in two equal size subsets of highly expressed (HE) and LE genes.

### Essentiality Data

We classified genes as essential or nonessential using *E. coli* data (Baba et al. 2006). An intraoperonic gene pair was considered essential (EE) if both genes were essential and nonessential (NN) when both genes were nonessential.

### Protein Level Analysis

To estimate *E. coli* K-12 MG1655 protein levels per cell, we used three proteomic experimental data sets (Lopez-Campistrous et al. 2005; Lu et al. 2007; Masuda et al. 2009). Data obtained from different experiments were normalized relative to their average values as the ones of the expression levels (discussed earlier). The relative difference (Δ_A_*_B_*) between concentrations of proteins encoded by two intraoperonic genes A (of concentration *C*_A_) and B (of concentration *C*_B_) was calculated as:

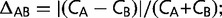

We used the median value of Δ to group proteins in two classes of respectively high and low concentration (supplementary fig. S3, Supplementary Material online). Pairs of intraoperonic genes belonging to the same class of concentration (both high or both low) were marked as having balanced protein concentrations (BAL). The remaining pairs were marked as having unbalanced protein concentrations (UNB).

### Analysis of Transcription Factors

Information for the predicted transcription factors for each genome was downloaded from the DBD transcription factor database (Charoensawan et al. 2013). We analyzed all organisms in our study for which there were predictions available of transcription factors: 49 α-proteobacteria, 35 β-proteobacteria, and 59 Firmicutes. We included in the analysis the nonredundant transcription factor protein families using UCLUST with a threshold of 80% (Edgar 2010).

### Phylogenetic-Independent Contrasts

For each clade (plus *E. coli* K-12 MG1655), we built a supermatrix concatenating the aligned protein sequences of the core genome (described earlier for the persistence analysis). The phylogenetic trees of the three clades were inferred from this supermatrix by maximum likelihood algorithms using PHYML (Guindon and Gascuel 2003) with the model LG + Γ(8) and default parameters. The trees were rooted using *E. coli* K-12 MG1655 as an outgroup. For each variable of interest, we carried out the phylogenetic-independent contrast (PIC) analysis (Felsenstein 1985) using the package ape (Paradis et al. 2004) for R software (R Development Core Team 2011). The analysis of contrasts showed in some clades some systematic outliers, caused by long internal branches in the tree. To include these points in the analysis without giving them unwarranted weight, we used nonparametric methods (Spearman rank) to examine the correlation between contrasts.

### Statistical Analysis

Correlations were analyzed by nonparametric Spearman rho association measure. The statistical differences between subsets (e.g., HE vs. LE) were assessed by *t*-paired tests. The Spearman partial correlation test was used to analyze the relation between the number of transcription factors and OCI while controlling for genome size. The tests of difference between correlation coefficients were computed from the Fisher’s *Z*-transformation (Kvam and Vidakovic 2007). All statistics were computed using R (R Development Core Team 2011).

## Results

### Associations between General Features of Operon Organization and Genome Features

We used two independent databases of operon predictions, ProOpDB and DOOR, to class pairs of adjacent genes as intraoperonic or interoperonic (see Materials and Methods). These analyses were done for 124 genomes of Firmicutes, 102 of α- and 63 of β-Proteobacteria (one genome per species). The analysis of both operon databases gave concordant results and we only present in the main text the results concerning ProOpDB, which reports the highest accuracy (Taboada et al. 2012; see supplementary text S1 [Supplementary Material online] for a comparison). Species in our data set are not independent statistical instances because they are related by a common evolutionary history. To correct for this effect, we retested all correlation analyses using PICs (see Materials and Methods; Felsenstein 1985; supplementary tables S3 and S4, Supplementary Material online). For simplicity, we indicate the results of these analyses in the main text only when they qualitatively contradict the significance of the Spearman nonparametric association test.

**Fig. 1.—:**
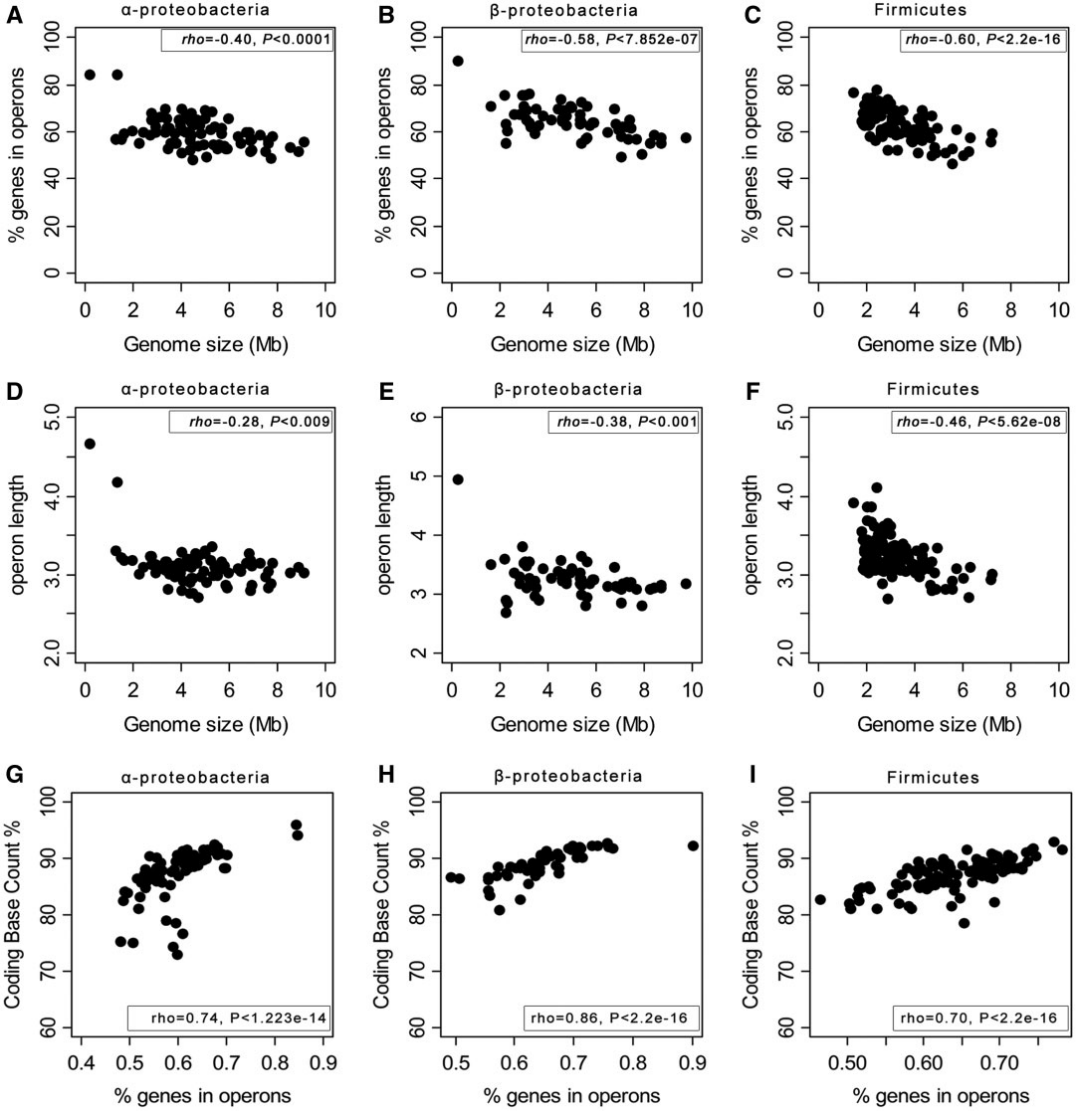
Association between genome size and the fraction of genes in operons, the length of operons and the density of coding sequences. Association between the fraction of genes in operons (*A–C*) and the length of operons as a function of genome size (*D–F*). Association between the fraction of genes in operons and the density of coding sequences (*G–I*). Results for α-Proteobacteria (*A*, *D*, *G*), β-Proteobacteria (*B*, *E*, *H*), and Firmicutes (*C*, *F*, *I*). Operon length was calculated as the average number of genes per operon for the whole genome operon predictions. Association between the variables was computed using the nonparametric Spearman rho.

The number of genes and the number of operons in genomes grows linearly with genome size (supplementary fig. S1, Supplementary Material online). This is expected given the high coding density of the genomes of prokaryotes (Mira et al. 2001). We found that a group of α-protebacteria shows a singular behavior in several plots regarding coding densities (supplementary fig. S4, Supplementary Material online). The group consists of organisms with an obligate intracellular lifestyle—either parasites or endosymbionts of the order Rickettsiales—ongoing extensive pseudogenization (Andersson and Andersson 2001). Many of these genomes have thus large intergenic regions. As mentioned earlier, intergenic distances are one of the key variables used to delimit operons. To avoid spurious definitions of operons caused by extensive pseudogenization, we excluded this group of 21 genomes of α-proteobacteria (supplementary table S5, Supplementary Material online) from further analyses. This had no qualitative impact on our results (supplementary fig. S5, Supplementary Material online). We found significant negative associations between genome size and the fraction of genes in operons and the average operon length in all clades (fig. 1*A*–*F*). We also found pervasive and strong positive correlations between the coding density of a genome and the average number of genes in operons (fig. 1*G*–*I*). This shows clear associations between genome size, operon organization and coding density. Notably, larger genomes tend to have relatively larger intergenic regions and fewer and shorter operons. This might be caused by stronger selection for operons in smaller genomes or simply by smaller intergenic distances within than between operons. To distinguish between these two hypotheses, we studied operon conservation.

### Operon Conservation and Genome Size

**Fig. 2.—:**
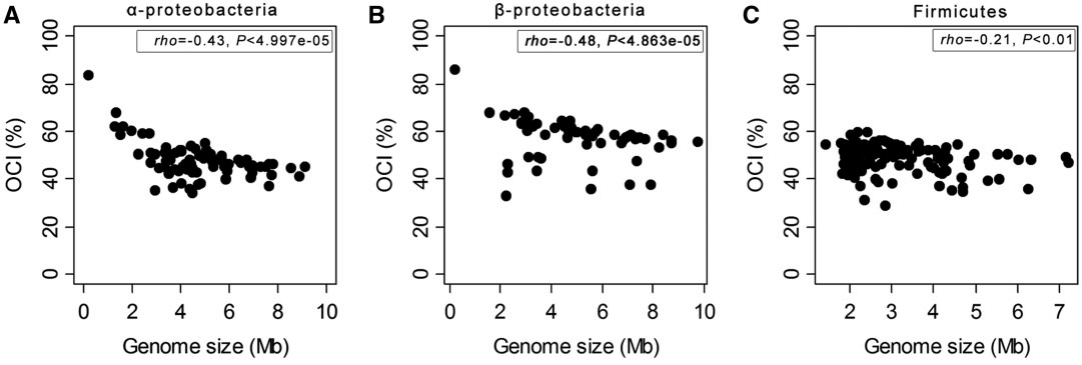
Association of the OCI with genome size. (*A*) α-Proteobacteria; (*B*) β-Proteobacteria; and (*C*) Firmicutes. Association between the variables was computed using the nonparametric Spearman rho.

We computed for each genome the OCI (see Materials and Methods). OCI is the fraction of intraoperonic pairs of adjacent genes in a genome having orthologs in one same operon in *E. coli*. OCI values are higher in β-proteobacteria (median = 0.57) as expected because this is the clade closest to *E. coli*. The other clades have lower medians and their respective ranks—Firmicutes (median = 0.50) and α-proteobacteria (median = 0.46)—are opposite to the expected given phylogenetic relatedness. Next, we examined the association between genome size and OCI. The three clades showed negative, statistically significant, correlations between operon conservation and genome size (fig. 2). Hence, larger genomes have fewer, shorter and less conserved operons than smaller genomes. To shed some light in the gene traits shaping operon conservation, we introduced in our analysis a series of biologically relevant variables such as gene essentiality, protein expression levels, and balanced protein concentration.

### Effects of Essentiality in Operon Conservation

**Fig. 3.—:**
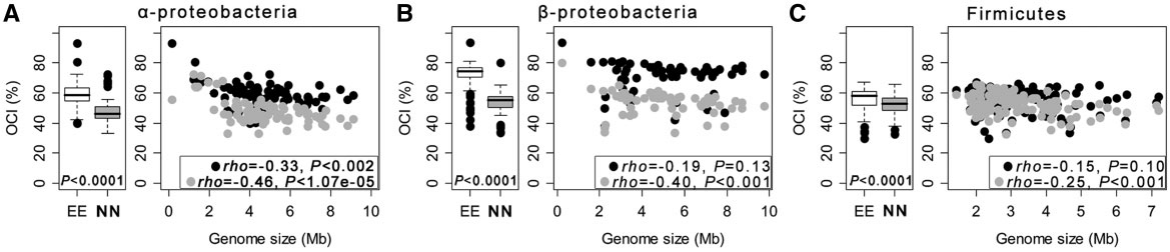
Association of the OCI with gene essentiality. Box plots indicate the distribution of OCI values per genome for pairs of intraoperonic essential (EE) and nonessential genes (NN). The box plots indicate the median (central line), the 25% and 75% percentiles (edges of boxes), the 1.5 interquartile ranges (whiskers), and the outliers (dots). Box plot associated *P* values correspond to the paired *t*-tests that means between the groups differ. Association between OCI and genome size for pairs of EE (black dots) and NN (gray dots) genes was computed using the nonparametric Spearman rho. For the α-Proteobacteria, the obligate intracellular cluster organisms were excluded of the correlation analysis (supplementary fig. S5, Supplementary Material online). Clades: (*A*) α-Proteobacteria, (*B*) β-Proteobacteria, and (*C*) Firmicutes.

Essential genes are usually defined as those whose inactivation prevents growth even in rich medium (Baba et al. 2006). The operons of *E. coli* overrepresent essential genes and those encoding them are highly conserved (Pal and Hurst 2004; Price, Huang, et al. 2005). Small genomes, which we have shown have more conserved operons, have a larger fraction of essential genes. We have thus tested the hypothesis that essentiality plays a role in the association between operon conservation and genome size. We classed genes as essential or nonessential using *E. coli* data (Baba et al. 2006), and tested whether pairs of essential genes (EE) are more often conserved in the same operon than pairs of nonessential genes (NN). This was true and statistically significant in all clades (*t*-paired tests, all *P* < 0.0001; fig. 3*A*–*C*), with stronger effects in α-Proteobacteria, then β-Proteobacteria and finally Firmicutes. We then tested how the presence of essential genes affected operon conservation in function of genome size. Both pairs of essential and nonessential genes tend to be less conserved in the operons of larger genomes (fig. 3*A*–*C*). Yet, the correlation is weak and often nonsignificant. These results not only show that operons with essential genes are more conserved, but also show that both operons with essential and nonessential genes are less conserved in large genomes.

### Expression Levels

**Fig. 4.—:**
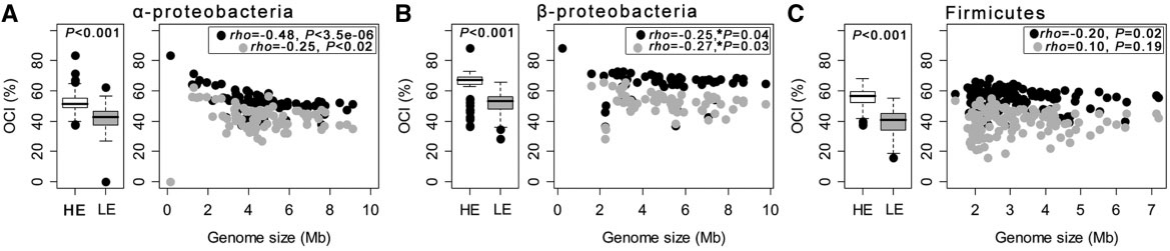
Association of the OCI with expression levels. Box plots indicate the distribution of OCI values per genome for intra-operonic pairs of HE and LE genes. The box plots indicate the median (central line), the 25% and 75% percentiles (edges of boxes), the 1.5 interquartile ranges (whiskers) and the outliers (dots). Box plot associated *P* values correspond to paired-*t* statistical tests that means differ. Association between OCI and genome size for pairs of HE (black dots) and LE (gray dots) genes was computed using the nonparametric Spearman rho. For the α-Proteobacteria, the obligate intracellular cluster organisms were excluded of the correlation analysis (supplementary fig. S5, Supplementary Material online). Clades: (*A*) α-Proteobacteria, (*B*) β-Proteobacteria, and (*C*) Firmicutes. (*) The associations for β-Proteobacteria become nonsignificant after control for phylogenetic contrasts (supplementary table S4, Supplementary Material online).

Highly expressed genes in bacteria are under stronger purifying selection and thus show higher codon usage bias (Gouy and Gautier 1982), encode less expensive amino acids (Akashi and Gojobori 2002), and evolve slower (Rocha and Danchin 2004). Similarly, gene organization of highly expressed genes is expected to be under stronger purifying selection because the cost of inefficient tuning of expression levels in highly expressed proteins is presumably higher. To study the effect of expression levels on operon conservation, we analyzed published transcriptomic data sets (see Materials and Methods). As expected, we found that pairs of HE genes in the same operon are more conserved than pairs of LE genes in all three clades (*t*-paired test on OCI values, *P* < 0.0001; fig. 4*A*–*C*). We then investigated the dependence of operon conservation on expression levels given genome size. We found that conservation of intraoperonic pairs of highly expressed genes show a systematic negative relationship with genome size (fig. 4*A*–*C*). Intraoperonic pairs of lowly expressed genes show less consistent patterns in the three clades and tend to be less conserved in larger genomes.

### Protein Dosage Effects

**Fig. 5.—:**
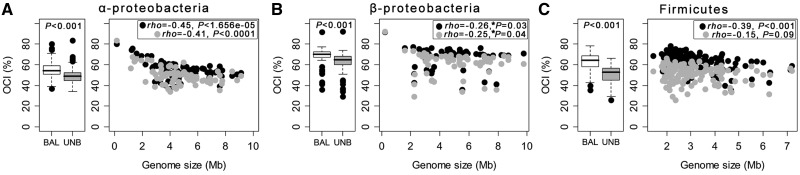
Association of the OCI with balanced protein concentration levels. Box plots indicate the distribution of OCI values per genome for pairs of genes encoding proteins with BAL and UNB concentrations in the cell. The box plots indicate the median (central line), the 25% and 75% percentiles (edges of boxes), the 1.5 interquartile ranges (whiskers) and the outliers (dots). Box plot associated *P* values correspond to paired *t*-tests that means differ. Association between OCI and genome size for pairs of genes encoding proteins with balanced (black dots) and unbalanced (gray dots) concentrations in the cell was computed using the nonparametric Spearman rho. For the α-Proteobacteria, the obligate intracellular cluster organisms were excluded of the correlation analysis (supplementary fig. S5, Supplementary Material online). Clades: (*A*) α-Proteobacteria, (*B*) β-Proteobacteria, and (*C*) Firmicutes. (*) The associations for β-Proteobacteria become nonsignificant after control for phylogenetic contrasts (supplementary table S4, Supplementary Material online).

Processes that depend on several proteins are often sensitive to changes in their relative concentration (Papp et al. 2003; Veitia 2004). If cotranscription allows minimization of gene dosage imbalance then one would expect operons to overrepresent pairs of genes encoding proteins with similar cell concentrations. Indeed, we observed in all three clades that OGPs encoding proteins with similar concentrations in *E. coli* cells were more conserved than OGPs with very different protein cell concentrations (see Materials and Methods, *t*-paired tests on OCI values, all *P* < 0.0001; fig. 5*A*–*C*). Posttranslation modifications and different protein turnovers may lead to different protein concentration in the cell even for genes expressed at similar levels in the same operon. Our results suggest that operons encoding proteins that are present at very different concentrations are under weaker selection. In both types of pairs of intraoperonic genes, there is a systematic trend for lower conservation in larger genomes, although trends are often weak as measured by the rho coefficient of association and its statistical significance (fig. 5*A*–*C*). Hence, the trends of operon conservation with genome size are similar for both classes and follow the general trend of weaker conservation in larger genomes.

### Transcriptional Regulation Complexity and Operon Conservation

**Fig. 6.—:**
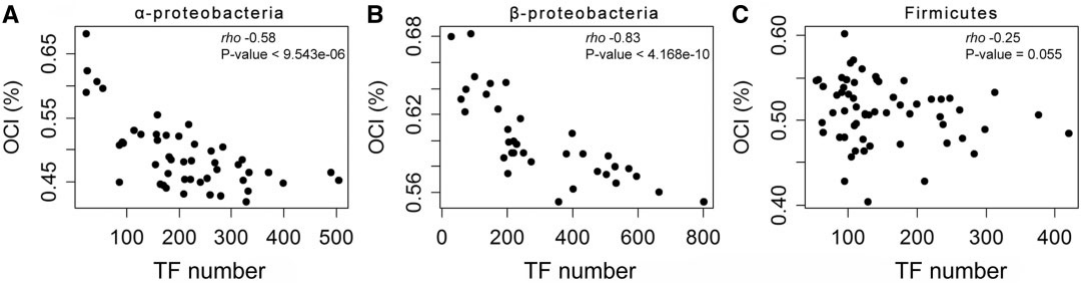
Association between the number of transcription factors, genome size, and the OCI. (*A–C*) For the three clades (α-Proteobacteria, β-Proteobacteria, and Firmicutes), the TF total number was inversely correlated with the OCI. Spearman correlation test (rho and *P* value) are indicated.

As mentioned earlier, the fraction of genes that encode transcription factors increases with the polynomial of genome size. Larger genomes encode complex regulatory networks and this might lead to decreased selection for operons in these bacteria for two reasons. First, it might be easier to encode a complex genetic network in monocystronic units where each gene is expressed independently. Second, the presence of a large number of transcription factors may lower the pressure to place coexpressed genes under the action of the same promoter. Conversely, small genomes often lack genes encoding transcription factors, which might increase the selection pressure for the presence of operons. To test this hypothesis, we analyzed the association between the number of transcription factors coded by each genome and its OCI, while controlling for genome size (see Materials and Methods). The number of transcription factors per genome and the OCI are inversely correlated (fig. 6; supplementary fig. S6, Supplementary Material online). They are also inversely correlated when controlling for genome size for each clade (Spearman partial correlation: α-proteobacteria *P* = 0.044, β-proteobacteria *P* = 0.087, and Firmicutes *P* = 0.065; fig. 6). Although the effects are weak, they are clearly significant using Fisher’s combined probability test (*P* = 0.01).

## Discussion

In this study, we confirmed previous suggestions that larger genomes tend to have fewer and shorter operons (Cherry 2003; Yus et al. 2009). Furthermore, we showed that larger genomes have less conserved operons. We confirmed that operons including highly expressed and essential genes are more conserved (Pal and Hurst 2004; Price, Huang, et al. 2005; Fang et al. 2008). Furthermore, we showed that operons with these traits are also less conserved in larger genomes. We cannot exclude the possibility that different gene repertoires in small and large genomes account for part of the effect we describe. Yet, lower operon conservation in larger genomes is not just caused by the overabundance in these genomes of nonessential, weakly expressed and recently acquired genes. Instead, all types of intraoperonic gene pairs, including the ones with essential genes that are nearly ubiquitous, are less conserved in larger genomes. Furthermore, the negative correlations of OCI with genome size are never significantly different between complementary traits (e.g., HE vs. LE) for a given clade (supplementary table S6, Supplementary Material online). This suggests that the correlation between OCI and genome size transcends these traits. Interestingly, we found that operons with genes expressing proteins at similar concentrations are more conserved. This conservation also decreases with increasing genome size.

Genome size is thought to correlate with a number of important traits such as effective population size (Kuo et al. 2009), the number of transcription factors (Konstantinidis and Tiedje 2004), and the frequency of horizontal gene transfer (Cordero and Hogeweg 2009). Genome size is also correlated with the abundance of transposable elements (Touchon and Rocha 2007). Abundance of transposable elements and horizontal gene transfer might increase the rates of chromosomal rearrangements and therefore lower operon conservation. On the other hand, the processes of genome reduction might also be associated with increased rearrangement rates and less efficient selection for genome organization (Moran and Mira 2001). Comparative genomics studies have observed that the conservation of gene order is under very strong purifying selection, with rearrangements having very small probabilities of fixation, and is not strongly correlated with genome size (Rocha 2006a). The conservation of operons over vast evolutionary timescales, despite high genome rearrangement rates and very frequent horizontal transfer, and the dependency of operon conservation on biologically relevant traits suggest that natural selection drives the evolution of operons. As genome size and operon structure and conservation are anticorrelated, we can use this information to assess the pertinence of the different evolutionary models of operon formation (tables 1 and 2).

**Table 1:** Summary of the Statistical Spearman Association Tests (Rho) between Genome Size and the OCI in Function of Different Traits

Data Sets	α-Proteobacteria	β-Proteobacteria	Firmicutes
	Rho (*P* Value)	Rho (*P* Value)	Rho (*P* Value)
Genome features			
% Coding density (%)	−0.24 (*P* < 0.03)	−0.37 (*P* < 0.007)	−0.34 (*P* < 0.0001)
% Genes in operons	−0.40 (*P* < 0.0001)	−0.58 (*P* < 0.001)	−0.60 (*P* < 0.002)
Operon length	−0.28 (*P* < 0.009)	−0.38 (*P* < 0.001)	−0.46 (*P* < 0.002)
OCI			
Total OGP	−0.43 (*P* < 0.0001)	−0.48 (*P* < 0.001)	−0.21 (*P* < 0.01)
EE	−0.33 (*P* < 0.002)	−0.19 (*P* = 0.13)	−0.15 (*P* < 0.0001)
NN	−0.46 (*P* < 0.0001)	−0.40 (*P* < 0.001)	−0.25 (*P* < 0.1)
HE	−0.48 (*P* < 0.0001)	−0.25 (*P* < 0.04)*	−0.20 (*P* < 0.02)
LE	−0.25 (*P* < 0.02)	−0.27 (*P* < 0.03)*	−0.10 (*P* = 0.19)
BAL	−0.45 (*P* < 0.0001)	−0.26 (*P* < 0.03)*	−0.39 (*P* < 0.001)
UNB	−0.41 (*P* < 0.0001)	−0.25 (*P* < 0.04)*	−0.15 (*P* = 0.09)

Note.—NS, nonsignificant (*P* > 0.05).

*Nonsignificance in the phylogenetic contrast analysis.

**Table 2:** Summary of the Statistical *t*-Paired Tests and Associated *P* Values When Testing the Difference in the OCI between Genes Pairs with Different Traits (EE vs. NN; HE vs. LE; BAL vs. UNB)

OCI	α-Proteobacteria	β-Proteobacteria	Firmicutes
	*t*-Paired (*P* Value)	*t*-Paired (*P* Value)	*t*-Paired (*P* Value)
EE	*P* < 0.001 ↓	*P* < 0.001 ↓	*P* < 0.001 ↓
NN			
HE	*P* < 0.001↓	*P* < 0.001 ↓	*P* < 0.001 ↓
LE			
BAL	*P* < 0.001 ↓	*P* < 0.001 ↓	*P* < 0.001 ↓
UNB			

Note.—(↓) Arrow direction goes from higher to lower conservation.

Linkage models consider that operons should be more conserved when encoding highly persistent (recombination and persistence models) or highly transferred genes (selfish model). The selfish model is at odds with the observation of less conserved operons in larger genomes (larger genomes endure higher rates of horizontal transfer) and with the higher conservation of operons with essential genes. However, this model is intended to explain best the existence of operons encoding highly transferred genes. It is possible that our emphasis on genes with homologs in *E. coli* leads to the dismissal of these genes. We therefore divided the genes of each genome in two categories: with and without orthologs in *E. coli*. We found a higher proportion of genes in operons for genes with orthologs in *E. coli* than for genes without (*t*-paired tests, all clades *P* < 0.001). This suggests that our approach is not biasing our conclusions regarding the selfish model. The other linkage models are consistent with high conservation of operons with essential and highly expressed genes. Operons with essential genes should be more conserved under the recombination model if linkage was under stronger purifying selection for pairs of these genes, which is plausible and has been observed in yeast (Pal and Hurst 2003). The persistence model predicts clustering of genes whose deletion is very deleterious as is the case for essential genes (Fang et al. 2008). As highly expressed genes are under strong purifying selection (Rocha 2006b), both models predict tight clustering of these genes. But these two models also predict increased selection for operons in larger genomes because these correspond to bacteria with higher effective population sizes (more efficient selection) and more gene transfer (increased selection for linkage). Our results point to the opposite trends, providing little evidence in favor of linkage models.

Models suggesting that operons reduce stochastic noise in gene expression are in agreement with the higher conservation of operons encoding proteins with balanced concentrations. They are also consistent with selection for operons with genes having higher fitness impact (essential genes; Wang and Zhang 2011). However, the high conservation of highly expressed operons does not fit these models, because these should be much less affected by stochastic noise in gene expression (Swain et al. 2002). These results do not preclude the possibility of selection for noise minimization within operons, for example, concerning the order of genes in operons and colinearity (Kovacs et al. 2009), or the relevance of protein interactions at lower expression levels (Ray and Igoshin 2012). Yet, they cast some doubts on the predominance of these effects on operon conservation.

Regulation models suggest that operons are selected for the intrinsic value of cotranscription (or cotranslation) as a gene expression strategy. These models fit well many of our observations. Indeed, one would expect stronger selection for tight gene regulation of expensive genes (highly expressed genes) and for genes with strong fitness effects (essential genes). Selection for operons containing proteins at similar cell concentrations is also expected under this model: operons with very unbalanced protein concentrations require unnecessary transcription of the less abundant protein to allow expression of the most abundant one. Hence, operons encoding proteins with similar concentration levels minimize transcription/translation costs.

All other things being equal, decreased selection for operons in larger genomes contradicts all available models, including the regulatory model, because selection should be more efficient in larger genomes. Yet, there is one difference between large and small genomes that deeply impacts the expectations of the regulatory model: transcription factors are proportionally much more abundant in larger genomes (Konstantinidis and Tiedje 2004). We have shown that there is a negative association between the abundance of transcription factors and operon conservation. This suggests that operons are under stronger selection in genomes with fewer transcription factors. The lack of transcription factors in smaller genomes might thus lead to increased selection of operons, as an alternative means of coregulating gene expression. Naturally, the number of transcription factors is just one of the variables shaping the complexity of genetic networks. These depend on the number and diversity of regulatory elements and of the elements regulating them, like environmental sensors. The latter are also much more abundant in large genomes (Galperin 2006).

We propose that smaller effective population sizes of bacteria harboring smaller genomes are compensated by increased selection for operons in these genomes. This increased selection pressure for operons might be caused by the fewer alternative ways of regulating gene expression that are available to these bacteria. Inversely, the very complex regulatory networks of large genomes might lead to lower selection for cotranscription. This might also explain why Eukaryotes, which typically have large complex regulatory networks, have so few operons even when their effective population sizes are very large.

## Supplementary Material

Supplementary text S1, figures S1–S7, and tables S1–S6 are available at *Genome Biology and Evolution* online (http://www.gbe.oxfordjournals.org/).

Supplementary Data
