# Supplementary material for: Natural Selection for Operons Depends on Genome Size
Source: Genome Biol Evol. 2013 Nov 6;5(11):2242–54. doi: 10.1093/gbe/evt174 (PMC3845653; doi:10.1093/gbe/evt174)
Supplement: Supplementary Data [file supp_5_11_2242__index.html]

Natural Selection for operons depends on genome size — Natural Selection for Operons Depends on Genome Size — Supplementary Data 

# Natural Selection for Operons Depends on Genome Size

## Supplementary Data

files

**Files in this Data Supplement:**

- Supplementary Data - doc file
- Supplementary Data - doc file
- Supplementary Data - doc file
- Supplementary Data - doc file
- Supplementary Data - doc file
- Supplementary Data - doc file
- Supplementary Data - doc file
- Supplementary Data - doc file
- Supplementary Data - xls file
- Supplementary Data - doc file
- Supplementary Data - doc file
- Supplementary Data - doc file
- Supplementary Data - doc file
- Supplementary Data - doc file
- Supplementary Data - doc file
