# Supplementary material for: Natural Selection for Operons Depends on Genome Size
Source: Genome Biol Evol. 2013 Nov 6;5(11):2242–54. doi: 10.1093/gbe/evt174 (PMC3845653; doi:10.1093/gbe/evt174)
Supplement: Supplementary Data [file supp_evt174_Figure_S1.doc]

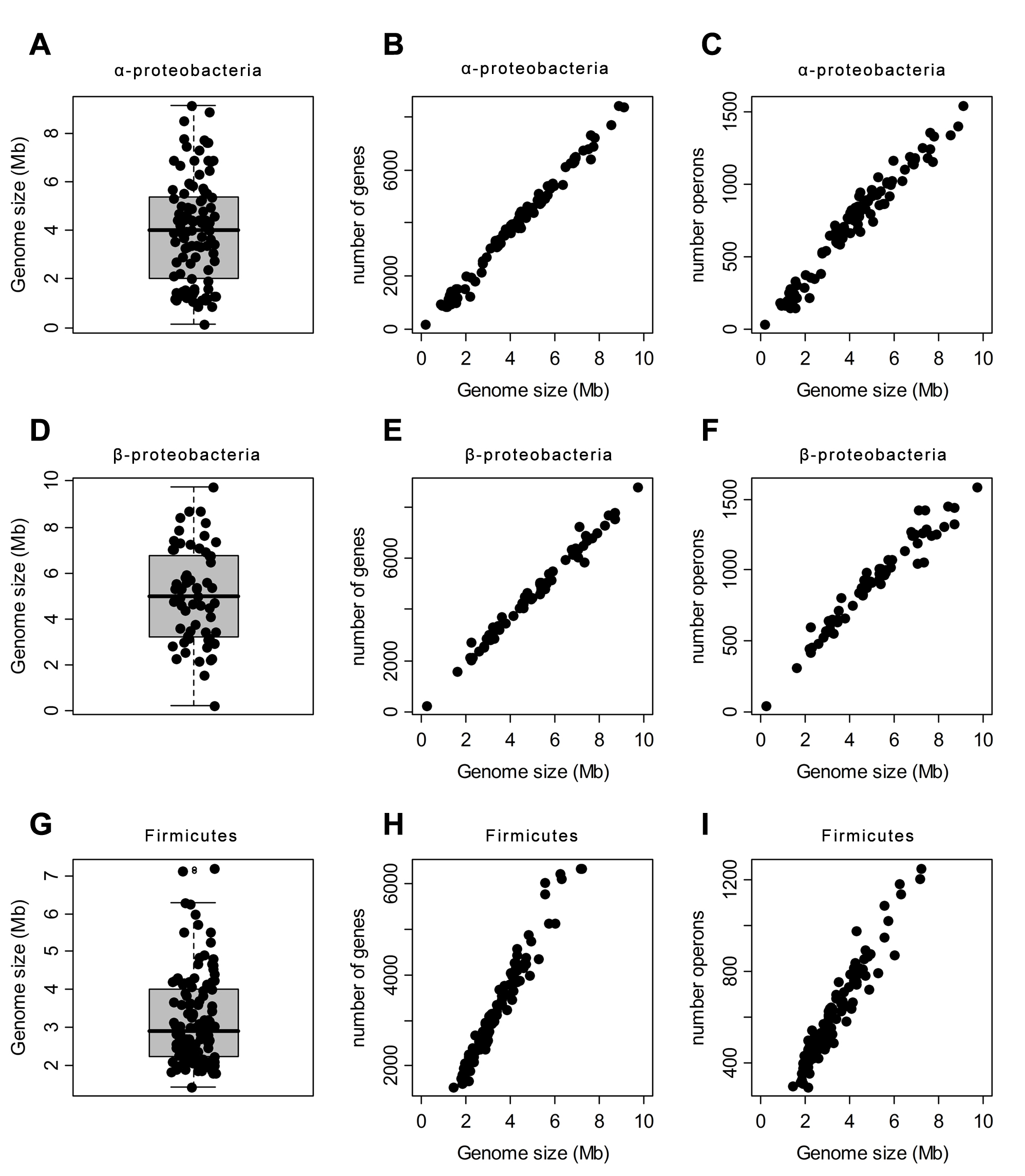


**Supplementary Figure S1.** Number of genes and operons in function of genome size.

For each clade (A-C) α-proteobacteria; (D-F) β-proteobacteria; (G-I) Firmicutes, we analyzed: the distribution of genome sizes (A, D, G), the number of genes (B, E, H), and the number of operons (C, F, I).
