# Supplementary material for: Natural Selection for Operons Depends on Genome Size
Source: Genome Biol Evol. 2013 Nov 6;5(11):2242–54. doi: 10.1093/gbe/evt174 (PMC3845653; doi:10.1093/gbe/evt174)
Supplement: Supplementary Data [file supp_evt174_Figure_S2.doc]

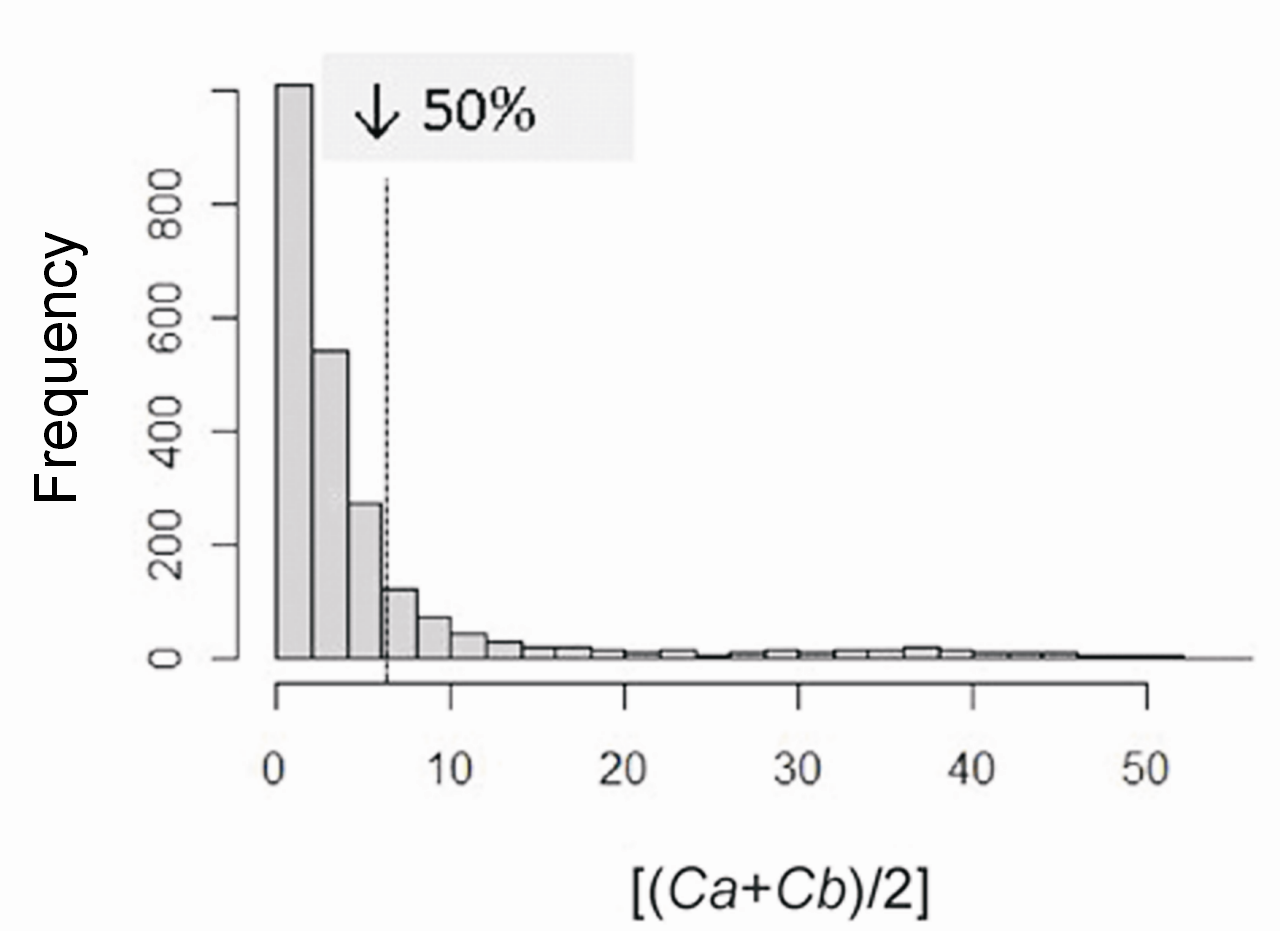


**Supplementary Figure S2.** Distribution of the expression levels for operon gene pairs in *E. coli*.

Distribution of the average expression levels of all operon gene pairs for *E. coli* based on the information of the mRNA concentration obtained from transcriptomic data.
