# Supplementary material for: Natural Selection for Operons Depends on Genome Size
Source: Genome Biol Evol. 2013 Nov 6;5(11):2242–54. doi: 10.1093/gbe/evt174 (PMC3845653; doi:10.1093/gbe/evt174)
Supplement: Supplementary Data [file supp_evt174_Figure_S3.doc]

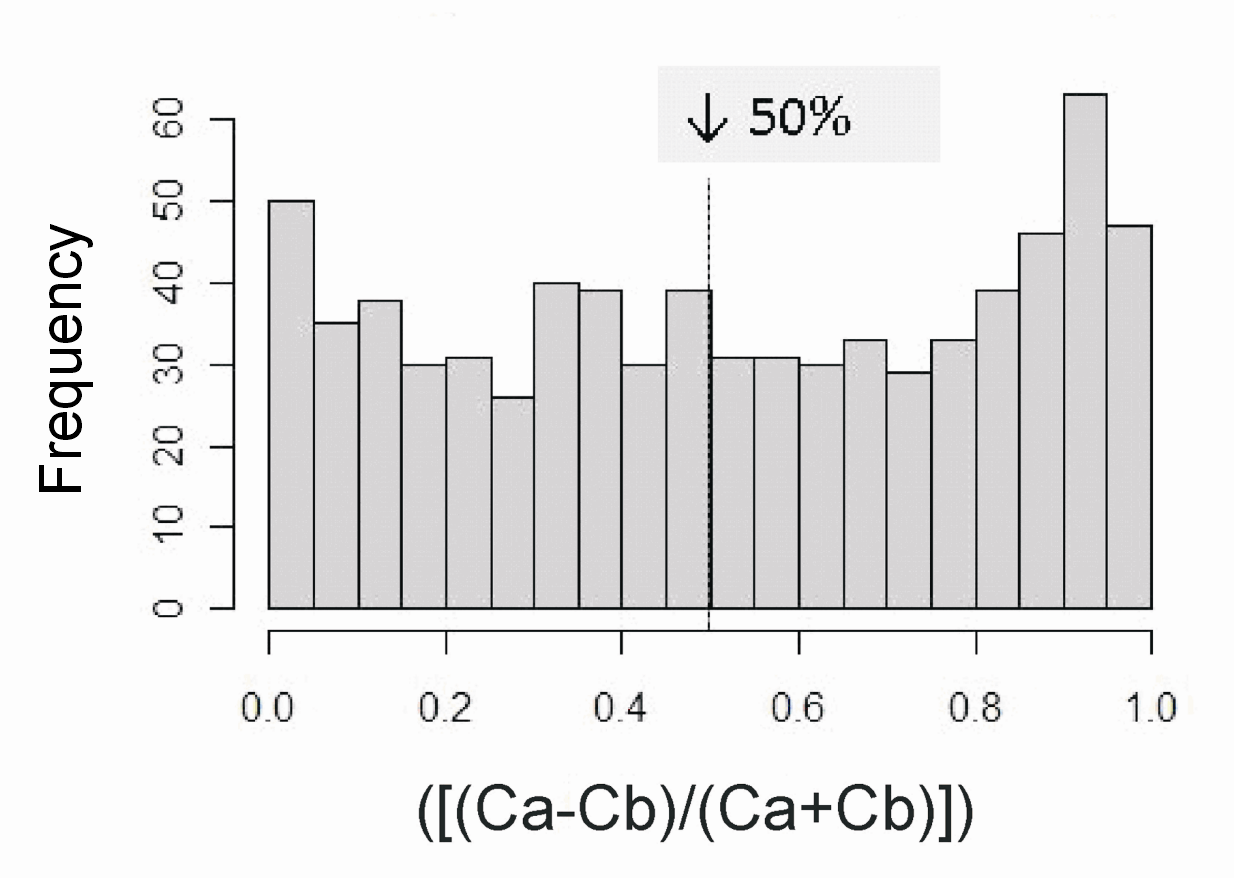


**Supplementary Figure S3.** Distribution of the differences in protein levels for operon gene pairs in *E. coli*.

The analysis was done for all *E. coli* operon gene pairs (*Ca* and *Cb*) for which there was proteomic data.
