# Supplementary material for: Natural Selection for Operons Depends on Genome Size
Source: Genome Biol Evol. 2013 Nov 6;5(11):2242–54. doi: 10.1093/gbe/evt174 (PMC3845653; doi:10.1093/gbe/evt174)
Supplement: Supplementary Data [file supp_evt174_Figure_S4.doc]

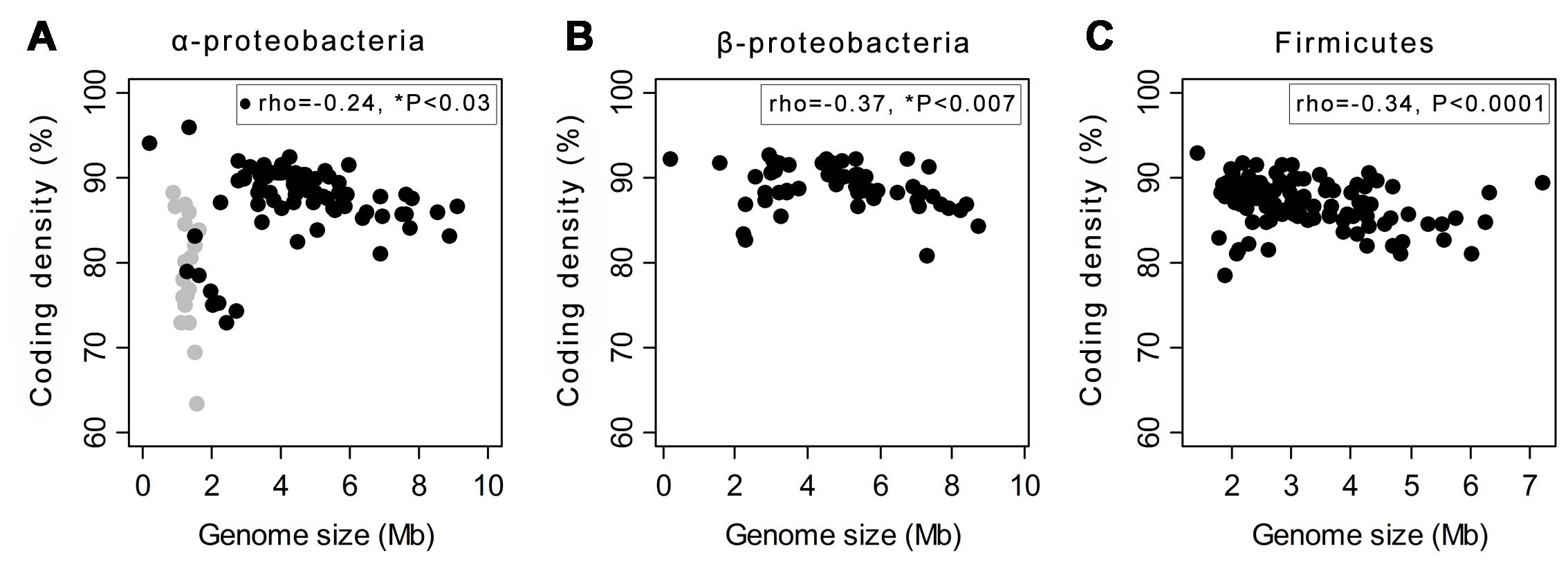


**Supplementary Figure S4.** Association between the density of coding sequences and genome size.

Association between the density of coding sequences and genome size for (A) α-Proteobacteria, (B) β-Proteobacteria and (C) Firmicutes. (*) The association between coding density and genome size is not significant (P>0.05) for α- and β-Proteobacteria after controlling for phylogenetic contrasts, respectively: α-Proteobacteria = -0.01 (NS) and β-Proteobacteria = -0.16 (NS). For the α-Proteobacteria, the obligate intracellular cluster organisms (grey points, Table S5) were excluded of the correlation analysis.
