# Supplementary material for: Natural Selection for Operons Depends on Genome Size
Source: Genome Biol Evol. 2013 Nov 6;5(11):2242–54. doi: 10.1093/gbe/evt174 (PMC3845653; doi:10.1093/gbe/evt174)
Supplement: Supplementary Data [file supp_evt174_Figure_S5.doc]

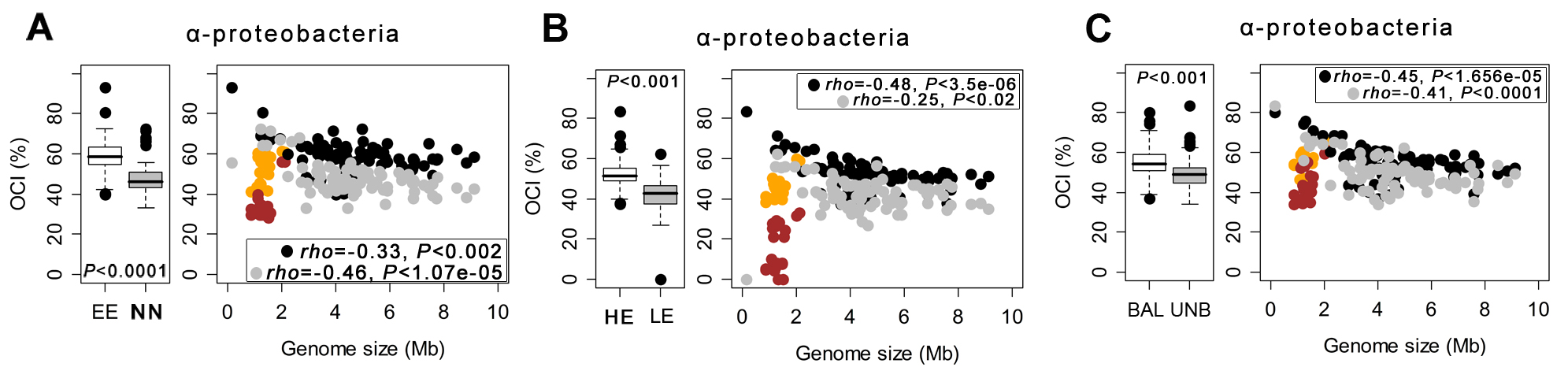


**Supplementary Figure S5.** Analysis of the α-Proteobacteria class without excluding the Rickettsiales.

**(A)** Analysis accounting for the gene essentiality: OCI values for the organisms within this group (Table S5) are indicated in orange (EE) and brown points (NN); **(B)** Analysis accounting for expression levels: OCI values for the organisms within this group (Table S5) are indicated in orange (HE) and brown points (LE); **(C)** Analysis accounting for the difference in protein concentrations: OCI values for the organisms within this group (Table S5) are indicated in orange (BAL) and brown points (UNB).
