# Supplementary material for: Natural Selection for Operons Depends on Genome Size
Source: Genome Biol Evol. 2013 Nov 6;5(11):2242–54. doi: 10.1093/gbe/evt174 (PMC3845653; doi:10.1093/gbe/evt174)
Supplement: Supplementary Data [file supp_evt174_Figure_S6.doc]

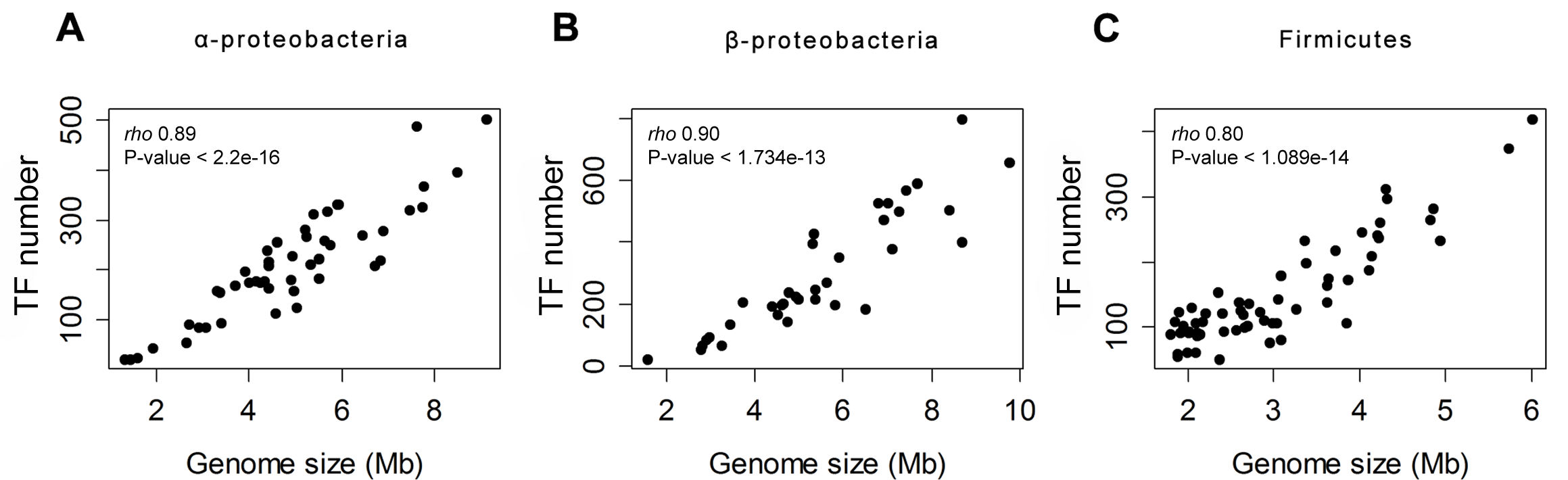


**Supplementary Figure S6.** Association between the number of transcription factors and genome size.

(A-C) For the three clades (α-Proteobacteria, β-Proteobacteria, Firmicutes) the total number of predicted transcription factors (TF) encoded in the genome was highly correlated with genome size. Spearman correlations (rho, P-value) are indicated.
