# Supplementary material for: Natural Selection for Operons Depends on Genome Size
Source: Genome Biol Evol. 2013 Nov 6;5(11):2242–54. doi: 10.1093/gbe/evt174 (PMC3845653; doi:10.1093/gbe/evt174)
Supplement: Supplementary Data [file supp_evt174_Figure_S7.doc]

**
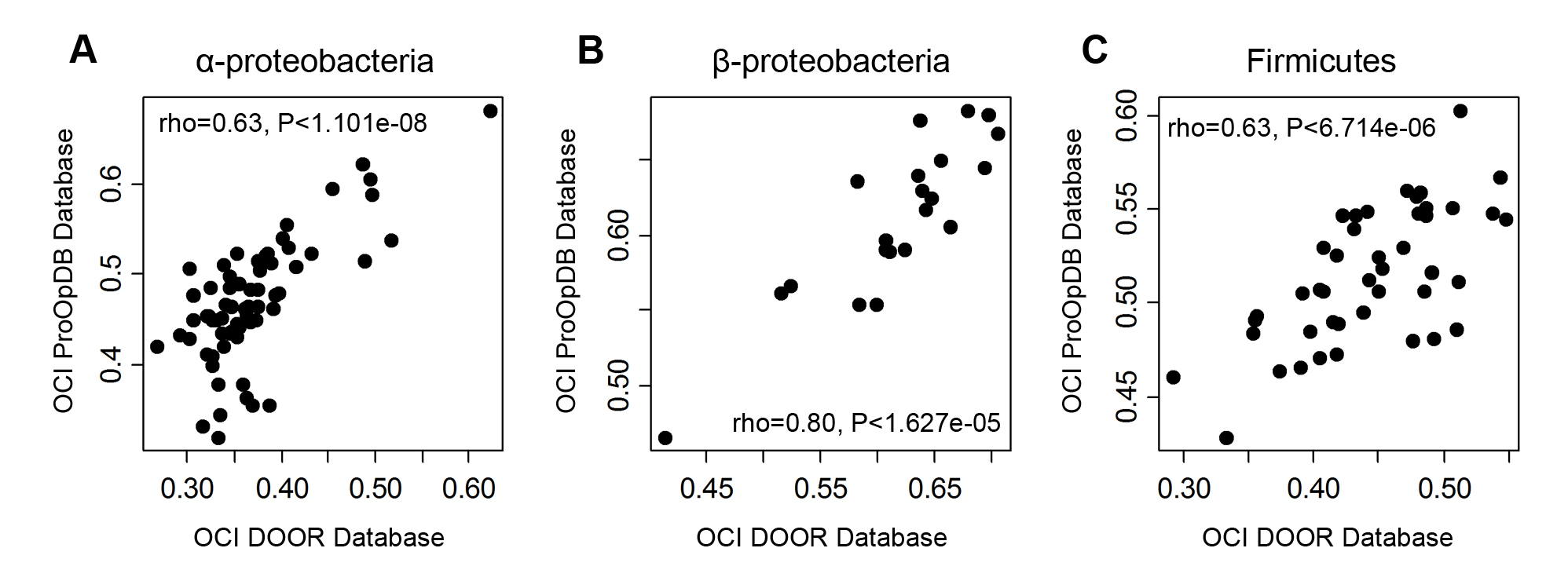
**

**Supplementary Figure S7.** Analysis of the association between OCI values obtained using two different databases; ProOpDB and DOOR.
