# Supplementary material for: Natural Selection for Operons Depends on Genome Size
Source: Genome Biol Evol. 2013 Nov 6;5(11):2242–54. doi: 10.1093/gbe/evt174 (PMC3845653; doi:10.1093/gbe/evt174)
Supplement: Supplementary Data [file supp_evt174_Supplementary_Material_Page.doc]

**Supplementary Materials for:**

**Natural Selection for operons depends on genome size**

Pablo A. Nuñez ^1, 5 *^, Héctor Romero ^2, 5^, Marisa D. Farber ^1^, Eduardo P.C. Rocha ^3, 4^

^1^ Instituto de Biotecnología, Instituto Nacional de Tecnología Agropecuaria (CICVyA-INTA), Buenos Aires, Argentina.

^2^ Laboratorio de Organización y Evolución del Genoma, Dpto. de Ecología y Evolución, Facultad de Ciencias / CURE, Universidad de la República, Uruguay.

^3^ Institut Pasteur, Microbial Evolutionary Genomics, 75015, Paris, France.

^4^ CNRS, UMR3525, 75015, Paris, France.

^5^ These authors contributed equally to this work

*Author for Correspondence: Pablo A. Nuñez, Instituto de Biotecnología, Instituto Nacional de Tecnología Agropecuaria, Buenos Aires, Argentina, (54)-11-4621-1447, [pb.nunez@gmail.com](mailto:pb.nunez@gmail.com).

Key words: Operons, Prokaryotes, Evolution.

# Supplementary Material

**Figure S1.** Number of genes and operons in function of genome size.

**Figure S2.** Distribution of the expression levels for operon gene pairs in *E. coli*.

**Figure S3.** Distribution of the differences in protein levels for operon gene pairs in *E. coli*.

**Figure S4.** Association between the density of coding sequences and genome size.

**Figure S5.** Analysis of the α-Proteobacteria class without excluding the Rickettsiales.

**Figure S6.** Association between the number of transcription factors and genome size.

**Figure S7.** Analysis of the association between OCI values obtained using two different databases; ProOpDB and DOOR.

**Tables S1.** Genome data, operons and Operon Conservation Index (OCI) in α-Proteobacteria, β-Proteobacteria and Firmicutes.

**Table S2**. Comparison of the correlation analyses (Spearman rho) that were done using the entire OGP dataset ("Total set") and using only non-overlapping gene pairs ("Reduced set") in the Firmicutes.

**Table S3.** Results of phylogenetic independent contrasts correlations between "genome size" and different coding and operon attributes (Figure 1).

**Table S4.** Results of phylogenetic independent contrasts correlations between the variables "genome size" and "ICO" for subsets delimited according to different traits.

**Table S5.** Obligate intracellular organisms from the order Rickettsiales (α-proteobacteria class) removed from the dataset.
