# Supplementary material for: Natural Selection for Operons Depends on Genome Size
Source: Genome Biol Evol. 2013 Nov 6;5(11):2242–54. doi: 10.1093/gbe/evt174 (PMC3845653; doi:10.1093/gbe/evt174)
Supplement: Supplementary Data [file supp_evt174_Table_S2.doc]

**Table S2.** Comparison of the correlation analyses (Spearman rho) that were done using the entire OGP dataset ("Total set") and using only non-overlapping gene pairs ("Reduced set") in the Firmicutes. The column Reduced vs. Total indicates the correlation between the two measures of OGP.

| **α-Proteobacteria** | | | | | | |
| --- | --- | --- | --- | --- | --- | --- |
|  | Reduced Set | | | Total Set | | Reduced vs Total* |
|  | *rho* | p-value | | *rho* | p-value | *r* value |
| Total OGP | 0.34 | < 0.005 | | -0.33 | < 0.005 | 0.988 |
| Essential Pairs (EE) | - 0.41 | < 0.0005 | | - 0.33 | < 0,002 | 0.908 |
| Non-essential Pairs (NN) | - 0.33 | < 0.005 | | - 0.46 | < 0,0001 | 0.907 |
| Highly Expressed (HE) | - 0.41 | < 0.0005 | | - 0.48 | < 0,0001 | 0.928 |
| Lowly Expressed (LE) | - 0.20 | NS | | - 0.25 | < 0,02 | 0.971 |
| Balanced (BAL) | - 0.49 | < 1E-5 | | - 0.45 | < 0,0001 | 0.939 |
| Unbalanced (UNB) | - 0.34 | < 0.005 | | - 0.41 | < 0,0001 | 0.923 |
| **β-Proteobacteria** | | | | | | |
|  | Reduced Set | | | Total Set | | Reduced vs Total* |
|  | *rho* | p-value | | *rho* | p-value | *r* value |
| Total OGP | -0.40 | < 0.005 | | -0.49 | < 0.0001 | 0.993 |
| Essential Pairs (EE) | -0.20 | NS | | -0.19 | NS | 0.977 |
| Non-essential Pairs (NN) | -0.43 | < 0.001 | | -0.40 | < 0.005 | 0.968 |
| Highly Expressed (HE) | -0.26 | < 0.05 | | -0.25 | < 0.05 | 0.985 |
| Lowly Expressed (LE) | -0.26 | < 0.05 | | -0.27 | < 0.05 | 0.974 |
| Balanced (BAL) | -0.26 | < 0.05 | | -0.26 | < 0.05 | 0.987 |
| Unbalanced (UNB) | -0.26 | < 0.05 | | -0.26 | < 0.05 | 0.989 |
| **Firmicutes** | | | | | | |
|  | Reduced Set | | | Total Set | | Reduced vs Total* |
|  | *rho* | | p-value | *rho* | p-value | *r* value |
| Total OGP | -0.28 | | < 0.005 | -0.2 | < 0.01 | 0.983 |
| Essential Pairs (EE) | -0.0004 | | NS | -0.15 | NS | 0.905 |
| Non-essential Pairs (NN) | -0.31 | | < 0.001 | -0.25 | < 0.001 | 0.906 |
| Highly Expressed (HE) | 0.03 | | NS | -0.20 | 0.02 | 0.921 |
| Lowly Expressed (LE) | 0.01 | | NS | 0.10 | NS | 0.930 |
| Balanced (BAL) | -0.34 | | < 0.001 | -0.39 | < 0.001 | 0.958 |
| Unbalanced (UNB) | -0.05 | | NS | -0.15 | NS | 0.939 |

* All P-values < 10e-15
