# Supplementary material for: Natural Selection for Operons Depends on Genome Size
Source: Genome Biol Evol. 2013 Nov 6;5(11):2242–54. doi: 10.1093/gbe/evt174 (PMC3845653; doi:10.1093/gbe/evt174)
Supplement: Supplementary Data [file supp_evt174_Table_S3.doc]

**Table S3.** Results of phylogenetic independent contrasts correlations between "genome size" and different coding and operon attributes (Figure 1).

| **Correlation** | **α-proteobacteria** | **β-proteobacteria** | **Firmicutes** |
| --- | --- | --- | --- |
| Genome size vs. % Fraction on genes in Operon | - 0.42 (P< 0.001) | - 0.35 (P< 0.02) | - 0.65 (P< 1e-10) |
| Genome size vs. Operon length | -0.26 (P<0.02) | - 0. 37 (P<0.01) | - 0.57(P< 1e-10) |
| Fraction of genes in operons vs. Density of coding sequences | 0.53(P<1e-6) | 0.49 (P<1e-4) | - 0.67(P< 1e-15) |
