# Supplementary material for: Natural Selection for Operons Depends on Genome Size
Source: Genome Biol Evol. 2013 Nov 6;5(11):2242–54. doi: 10.1093/gbe/evt174 (PMC3845653; doi:10.1093/gbe/evt174)
Supplement: Supplementary Data [file supp_evt174_Table_S4.doc]

**Table S4**. Results of phylogenetic independent contrasts correlations between the variables "genome size" and "ICO" for subsets delimited according to different traits.

| **Subset (OGP)** | **α-proteobacteria (without Rickettsiales)** | **β-proteobacteria** | **Firmicutes** |
| --- | --- | --- | --- |
| **Figure 2** | | | |
| All | - 0.47 (P<10e-6) | - 0.32 (P<0.01) | - 0.23 (P<0.01) |
| **Figure 3** | | | |
| Essential (EE) | - 0.22 (P<0.05) | - 0.14 (NS) | - 0.19 (P<0.05) |
| Non-essential (NN) | - 0.52(P<10e-6) | - 0.30 (P<0.01) | - 0.28 (P<0.01) |
| **Figure 4** | | | |
| Highly-expressed (HE) | - 0.38 (P<0.001) | - 0.12 (NS) | - 0.38 (P<0.001) |
| Lowly-expressed (LE) | - 0.30 (P<0.01) | - 0.22 (NS) | - 0.30 (P<0.01) |
| **Figure 5** | | | |
| Balanced (BAL) | - 0.33 (P<0.01) | - 0.10 (NS) | - 0.36 (P<0.001) |
| Unbalanced (UNB) | - 0.32 (P<0.01) | - 0.17 (NS) | - 0.20 (P<0.05) |
