# Supplementary material for: Natural Selection for Operons Depends on Genome Size
Source: Genome Biol Evol. 2013 Nov 6;5(11):2242–54. doi: 10.1093/gbe/evt174 (PMC3845653; doi:10.1093/gbe/evt174)
Supplement: Supplementary Data [file supp_evt174_Table_S5.doc]

**Table S5.** Obligate intracellular organisms from the order Rickettsiales (α-proteobacteria class) removed from the dataset.

| **Taxonomy** | **Genome size (MB)** |
| --- | --- |
| *Neorickettsia sennetsu* Miyayama | 0.85 |
| *Neorickettsia risticii* Illinois | 0.87 |
| *Wolbachia wBm* endosymbiont of *Brugia malayi* TRS | 1.08 |
| *Rickettsia typhi* Wilmington (ATCC VR-144) | 1.11 |
| *Rickettsia prowazekii* Madrid E | 1.11 |
| *Rickettsia canadensis* McKiel | 1.15 |
| *Ehrlichia chaffeensis* Arkansas | 1.17 |
| *Anaplasma marginale* St. Maries | 1.19 |
| *Anaplasma centrale* Israel | 1.20 |
| *Rickettsia akari* Hartford | 1.2 |
| *Wolbachia sp. wMel* endosymbiont of *Drosophila melanogaster* | 1.26 |
| *Rickettsia rickettsii* Iowa | 1.26 |
| *Ehrlichia canis* Jake | 1.31 |
| *Rickettsia massiliae* MTU5 | 1.37 |
| *Anaplasma phagocytophilum* HZ | 1.47 |
| *Wolbachia pipientis* endosymbiont of *Culex quinquefasciatus* Pel | 1.48 |
| *Ehrlichia ruminantium* Welgevonden | 1.51 |
| *Rickettsia bellii* OSU 85-389 | 1.52 |
| *Rickettsia felis* URRWXCal2 | 1.58 |
| *Orientia tsutsugamushi* Ikeda | 2.00 |
| *Orientia tsutsugamushi* Boryong | 2.12 |
