# Supplementary material for: Natural Selection for Operons Depends on Genome Size
Source: Genome Biol Evol. 2013 Nov 6;5(11):2242–54. doi: 10.1093/gbe/evt174 (PMC3845653; doi:10.1093/gbe/evt174)
Supplement: Supplementary Data [file supp_evt174_Table_S6.doc]

**Table S6.** P-values of the statistical tests to assess the significance of the difference between coefficients of correlation between operons with opposed traits. The test was done using a z-Fisher transformation.

| Comparison | α-proteobacteria | β-proteobacteria | Firmicutes |
| --- | --- | --- | --- |
|  | P-value | P-value | P-value |
| Essential (EE) | P=0.86 | P=0.90 | P=0.79 |
| Non-essential (NN) |  |  |  |
| High-expression (HE) | P=0.03* | P=0.55 | P=0.21 |
| Low-expression (LE) |  |  |  |
| Balanced (BAL) | P=0.36 | P=0.48 | P=0.02* |
| Unbalanced (UNB) |  |  |  |
| (*) Non-significant after Bonferroni correction for multiple tests. | | | |
