# Supplementary material for: Natural Selection for Operons Depends on Genome Size
Source: Genome Biol Evol. 2013 Nov 6;5(11):2242–54. doi: 10.1093/gbe/evt174 (PMC3845653; doi:10.1093/gbe/evt174)
Supplement: Supplementary Data [file supp_evt174_Text_S1.doc]

**Text S1. Comparison between ProOpDB and DOOR.**

The analysis comparing the OCI for each genome included in both datasets provided a fairly strong correlation between the different methods used to predict operons (Figure S7). The two methods provided qualitatively similar results. Using DOOR we found similar differences in conservation between essential and non-essential (P<0.01 for the three clades), highly and lowly-expressed (P<0.01 for the three clades) and balanced and unbalanced operons (P<0.01 for the three clades). Also, essential, highly expressed and balanced operons were found to be less conserved in larger genomes using the DOOR database, although the P-values were sometimes larger than 0.05, especially for β-proteobacteria which has the lowest sample size.
